# Role of the Patient’s History of Vestibular Symptoms in the Clinical Evaluation of the Bedside Head-Impulse Test

**DOI:** 10.3389/fneur.2017.00051

**Published:** 2017-02-20

**Authors:** Christoph Helmchen, Julia Knauss, Peter Trillenberg, Anita Frendl, Andreas Sprenger

**Affiliations:** ^1^Department of Neurology, University of Luebeck, Luebeck, Germany; ^2^Institute of Psychology II, University of Luebeck, Luebeck, Germany

**Keywords:** vestibulo-ocular reflex, head-impulse test, sensitivity, patient’s history, bedside examination

## Abstract

**Objective:**

Our aim was to identify the role of the investigators’ knowledge of the patient’s history of vestibular symptoms (PVH) in the clinical evaluation of the bedside head-impulse test (bHIT). We hypothesized that this knowledge will reduce uncertainty and improve bHIT accuracy when compared to quantitative analysis of the vestibulo-ocular reflex by video head-impulse test (vHIT).

**Methods:**

We looked for changes in the clinical assessment of the bHIT in 594 consecutive patients before and after taking PVH. bHIT was performed by 12 clinical neurologists with various clinical experience in neuro-otological diseases (novices to long-standing experts). vHIT was analyzed by four experts being blinded for the patients’ clinical presentation and history of symptoms. The confidence of bHIT and vHIT was rated (0–100%).

**Results:**

One hundred fifty-four (15%) of 1,030 bHIT of all eligible patients (*n* = 515) were rated pathological. Thirty-five (22.7%) of them were rated bilateral vestibulopathies. Sensitivity of bHIT reached 56.3%, its specificity 92.4%; the positive predictive value (PPV) was 41.5% and the negative predictive value 95.7%. These data did not differ between bHIT before and after PVH. bHIT after PVH (post-bHIT) differed from pre-bHIT in 44.3%, usually with regard to the level of confidence but also in polarity (5%). The accuracy of changes in bHIT depended on the direction of change: a “normal” post-bHIT was correct in 92.3% while only 39.8% of pathological post-bHIT were pathological on vHIT. However, sensitivity of a pathological post-bHIT depended on the clinical experience in taking PVH and bHIT: the PPV was 20.5% in novices as compared to 69.6% in experts.

**Conclusion:**

The study shows that PVH changes the certainty and/or polarity of the clinical evaluation of bHIT. Unlike expected, the increase in confidence in post-bHIT is associated with a consistently high specificity but no increase in sensitivity. Accuracy of changes in post-bHIT depends on the investigators’ clinical experience: it increases only in experts but not novices. Since novices show only a poor PPV and moderate sensitivity of bHIT, pathological bHITs should be controlled by vHIT, even in patients with a positive PVH. By contrast, confirmed normal post-bHIT is usually correct.

## Introduction

The vestibulo-ocular reflex (VOR) stabilizes retinal images during head motion. It can be clinically assessed by the bedside head-impulse test (bHIT) (1). There are several quantitative recording methods, scleral search coil, and head mounted-video-oculography devices (2, 3), which can precisely identify abnormal VOR deficits that may be missed by the bHIT: covert compensatory and anti-compensatory saccades during the head impulse movement (4). According to these state of the art recordings of the high frequency VOR, there is a reasonable but moderate sensitivity (63–72%) (5), in particular in patients with mild vestibular hypofunction (6, 7). Some patients may exhibit only refixation saccades possibly reflecting a previous VOR deficit (8). This may account for the moderate sensitivity of bHIT which differs from video head-impulse test (vHIT) in about 30% (9). History of patients’ symptoms, specifically vestibular-related symptoms, is crucial in determining the clinical vestibular disease entity (10) and is—if not searched for—a major cause of false diagnostic assignments. Patients with vestibular hypofunction usually complain about unsteadiness on stance and gait, lateropulsion, and blurred vision or oscillopsia, particularly during rapid head movements.

The aim of this prospective double-blind study was to investigate if symptoms of vestibular hypofunction in the patient’s history change the clinical assessment of the bHIT. Vestibular-related symptoms may lead to a bias in the clinical assessment of bHIT provoking false positive or false negative results.

We hypothesized that the sensitivity of bHIT after knowing the patient’s history will improve both with respect to its positive predictive value (PPV) and negative predictive value (NPV). bHIT was rated pathological and negative from 0 to 100% certainty and compared to quantitative vHIT which was recorded by video-oculography (EyeSeeCam^®^) and analyzed anonymously by experienced neuro-otologists. Sensitivity, specificity, PPV, and NPV of bHIT were determined.

## Patients and Methods

### Participants

We tested 594 consecutive outpatients (251 males, age range: 17–94; 58.6 ± 16.5 years) in our tertiary vertigo center at the University of Luebeck (Department of Neurology) before and after taking patient’s history. Twelve independent clinical neurologists of various expertise levels (novices to experts) participated in the study to take clinical experience of searching for vestibular-related symptoms and clinical skills in bHIT into account (5). Patients presented with the chief complain of vertigo, dizziness, or unsteadiness which could be episodic or chronic (>2 months) but we did not include patients with acute vestibular syndromes. Patients were excluded for the following reasons: vHIT could not be conducted (tolerance, disease-related restriction of head movements), decreased visual acuity, eye muscle disease, denial of informed consent in participating in the study, or discrepancies between blinded independent investigators of the vHIT, i.e., only those were included in which raters showed consistent pathological or normal assignments. Seventy-nine patients were excluded. Accordingly, 515 patients entered the study. Final clinical diagnoses are listed in Table 1.

**Table 1:** **Range and frequency of clinical diagnoses in 515 patients with dizziness (*n*, %)**.

Somatoform dizziness	153	29.7%
Benign paroxysmal positioning vertigo	144	28.0%
Vestibulopathy	64	12.4%
Unilateral	42	8.1%
Bilateral	22	4.3%
Menière’s disease	41	8.0%
Proprioceptive ataxia	36	7.0%
Cerebellar ataxia	20	3.9%
Central vestibular syndromes	15	2.9%
Unclassified	14	2.7%
Migraine	12	2.3%
Vestibular paroxysmia	8	1.6%
Central gait disorders	6	1.2%
Vestibular schwannoma	2	0.4%

### Study Protocol

The study was approved by the Ethics Committee of the University of Luebeck. Each participant provided informed consent in accordance with the Declaration of Helsinki. The first bHIT (pre-bHIT) was performed immediately when the patient entered the examination room. At this point, the clinical investigator had no documented or clinical information about the patient yet. The patient was informed that this first examination is meant to be a vestibular test of sensitivity of clinical skills. Afterward patients were asked for a history of symptoms and signs of past or present vestibular hypofunction in a standardized manner, i.e., head movement-related dizziness, visual blurring and/or oscillopsia, lateropulsion, or illusionary tilt perceptions (PVH). Either of these symptoms would be taken as positive, i.e., indicative for previous or current vestibular failure. Otherwise, PVH was classified negative. This included patients with a history of benign paroxysmal positional vertigo unless they presented additional symptoms of vestibular hypofunction. After PVH, the second bHIT was performed in the same manner (post-bHIT).

Twelve clinical investigators participated in the study. They had various clinical experiences with vertigo patients and with neuro-otological examinations: (i) novices, with a minimum of 3 months at the university vertigo clinic (*n* = 9), (ii) mid-level clinicians (2 years at the vertigo clinic, *n* = 2), and (iii) expert (>20 years at the vertigo clinic, *n* = 1). All of the investigators had at least 3 years of experience in general neurology.

Quantitative data of vHIT were analyzed by four experts with longstanding experience in the electrophysiological evaluation of vestibular function tests being blinded for the patients’ name, clinical presentation, and history of symptoms (see analysis).

### Bedside Head-Impulse Test (bHIT)

The bHIT was conducted as described previously (1, 11, 12). We confined the data collection for this study to the horizontal semicircular canals as they have the largest VOR gain and investigators are familiar with their clinical examination. In short, the patient was asked to fixate the tip of the investigator’s nose and to maintain this target fixation on short (10–20°) but unpredictable rapid head thrusts either to the right or to the left. The patient’s head was tilted slightly downward to adjust the horizontal canal to the stimulation plane. While investigator and the patient were facing each other, the investigator reached out with straight arms to put his hands on both sides of the patient’s head across the ears with the thumb elevated next to eye brows. The patient was instructed about the passive head rotations, then pilot movements were performed to reduce anxiety-related neck muscle resistance and afterward at least six unpredictable head thrusts were conducted randomly to either side. The bHIT was called pathological when the eyes repetitively drifted off the target to the side in which the head was moved and when centripetal catch up saccades were visible on the lesion side to resume fixation of the investigator’s nose, either during or immediately after the head thrust, on one [unilateral vestibular failure (UVF)] or both sides [bilateral vestibular failure (BVF)]. The clinical investigators evaluated pathological vs. normal bHIT and indicated their level of confidence (certainty range from 0 to 100%). The investigators did not know of any patient’s oculomotor or vestibular signs before post-bHIT. This allowed to detect changes in the assessment of bHIT before and after taking the patient’s history, i.e., bHIT assessment could reach a higher or lower level of confidence after knowing the patient’s history or even change its polarity (conversion from a pathological to normal bHIT or *vice versa*).

### Video Head-Impulse Test

The head-impulse test of all participants was recorded by a head-mounted video-oculography (11, 13–15). Eye and head movements were recorded by a digital video camera and six-degree-of-freedom inertial sensors, the EyeSeeCam^®^ HIT System (Autronics, Hamburg, Germany), at a sampling rate of 220 Hz (4). Eye position was detected using a pupil detection algorithm. Rubber bands were individually adapted to prevent video slippage during vHIT. Only the left eye was recorded monocularly. Calibration was performed prior to recording by asking the participant to repetitively fixate nine LED targets in random order projected on the screen in front of him at eye level from glasses-mounted laser. The participant was sitting on a chair in front of the investigator while he was fixating a laser target at a distance of 100 cm. He was asked to continue fixating this target during HITs which were unpredictable for direction and onset. The investigator was standing behind the patient and delivered repetitive (at least 10, each side) passive and rapid (3,000–4,500°/s^2^) head rotations (HIT) of small amplitude (10–15°) in the plane of the horizontal semicircular canals. Prior to recording, the head rotations were demonstrated slowly to improve the compliance of the participant. The investigators’ hands on both sides of the patient’s head did not touch the fixating rubber bands to prevent video slippage. Head velocity traces of VOR responses were automatically calculated and immediately displayed which allowed immediate correction, if required. Caution was made to include only HIT trials with peak head velocity exceeding 200°/s. Invalid head impulses contaminated by blinks were excluded.

### HIT Analysis and Statistical Analysis

Analysis of vHIT was performed by four different expert neuro-otologists blinded for the patient’s name, patient’s history, and clinical findings. Due to the known technical shortcomings of using a single VOR gain cutoff, in particular in moderate to modest VOR impairments (7, 16), vHIT was graded “pathological” or “normal” based on four analytical vHIT criteria: (i) gain at a narrow time interval, i.e., 60 ms (±10 ms) see EyeSeeCam (17) after head movement onset, (ii) slope derived from the ratio of robust linear regression of eye and head velocity between setpoints of head movement onset (head velocity >10°/s) and 90% of peak head velocity, i.e., the “Halmagyi gain” (18), (iii) profiles of individual head and eye velocity traces, and (iv) compensatory corrective saccades (overt and covert) (9). While overt saccades are visible on clinical examination after head thrust termination, covert refixation saccades have a shorter latency and occur while the head is still moving during HIT. Therefore, they are difficult to be detected at the bedside examination. A pathological VOR could be diagnosed on the basis of a VOR asymmetry, a slight VOR gain reduction with synchronized corrective (compensatory) saccades, or reduced or even truncated eye velocity traces. The rationale for this approach is our experience that the automatically detected VOR gain as the sole marker of VOR performance bears a considerable risk of false VOR assessments. One needs to identify pseudo-corrective saccades, mini and makro-blinks, “wrong-way” saccades, wrong eye direction slow phases due to patient’s inattention, phase shifts, double peaks, inappropriate high gain, and head overshoots to reduce the risk of false evaluations (11, 19).

In none of the participants, vestibular failure was diagnosed with a VOR gain >0.85. The four independent raters also evaluated their assignment (pathological vs. normal) on a confidence range from 0 to 100%. Only consistent assignments (i.e., all normal or all pathological) of all four raters were used for the comparison of bHIT and vHIT, irrespective of the criterion (1–4) they used for the decision and their level of confidence (0–100%). This reduced the number of eligible patients to 515. This resulted in 1,030 vHIT for the left and right side. All raters (except for one, 1 year) had long-standing (>3 years) experience in evaluating vHIT. They were blinded for the assessment of fellow raters. Finally, the raters had to indicate which of the four criteria was most suitable in determining the pathological state of VOR responses. Finally, in an independent analysis step, this method was compared with a commonly used single cutoff criterion of a pathological VOR gain, i.e., <0.7, which resulted from calculations of mean VOR gain of healthy subjects ± 2 SD units which incorporate 95% of the population (7, 20, 21). The vHIT ratings of the four raters were compared with the two corresponding bHIT (pre- and post-bHIT) for each side separately (left, right). This allowed to analyze sensitivity and specificity of pre- and post-bHIT as well as to determine the PPV and NPV. This was analyzed for all clinical investigators (mean) and for the three groups of different clinical neuro-otological expertise separately.

### Statistical Analysis

Sensitivity was calculated by the ratio of pathological bHITs in pathological vHITs vs. all pathological vHITs. Specificity was calculated by the ratio of negative (normal) bHITs in negative vHITs vs. all negative vHITs. The PPV was calculated by the ratio of pathological bHITs in all pathological vHITs vs. all pathological bHITs, the NPV by the ratio of negative bHITs in all negative vHITs vs. all negative (normal) bHITs.

The inter-rater reliability of pre- and post-bHIT was estimated by the Krippendorff’s alpha (22). Mean values are given with SE unless otherwise stated.

## Results

A total of 515 patients with 1,030 bHITs (left and right side) were compared with quantitative vHIT. One hundred fifty-four (15%) of all 1,030 bHIT were clinically rated pathological [73 on the right, 81 on the left side; 35 (22.7%) of them were bilaterally pathological] in the post-bHIT after taking PVH. Thus, the majority of bHIT were rated normal (*n* = 876, 85%).

Using a common VOR cutoff gain of <0.7 to determine pathological responses (7, 16), 58 of all patients were classified pathological on vHIT, 38 of them showed unilateral lesions (UVF) (29 left-sided: mean gain left 0.52; right 0.9; 9 right-sided: mean gain right 0.51; left 1.0; mean gain of both sides: 0.5 ± 0.04) and 20 patients had bilateral failure (BVF; mean gain left = 0.37, right = 0.44). Accordingly, 78 vHIT were pathological in these 58 patients with UVF and BVF. Except for five patients all of them were classified pathological by the common ratings of the four expert raters which we preferred for the further analysis. This high concordance between the two methods is reflected by a high sensitivity of 93.6%, a specificity of 97.7%, a PPV of 93.6%, and a NPV of 99.5% (see below).

By comparing bHIT and vHIT, sensitivity of pre-bHIT was 58.7%, its specificity 91.3%; the PPV 40.3% and the NPV 95.7%. After taking the patient’s history, the sensitivity of post-bHIT reached 56.3%, its specificity 92.4%; the PPV was 41.5% and the NPV 95.7%. Thus, there was no change after knowing PVH when all investigators were analyzed together. Since correct and false changes could counterbalance each other we looked at the types of pre- vs. post-bHIT changes.

### Changes in bHIT Evaluation (Comparison Pre- vs. Post-bHIT)

#### Frequency of Changes

**Figure 1:**
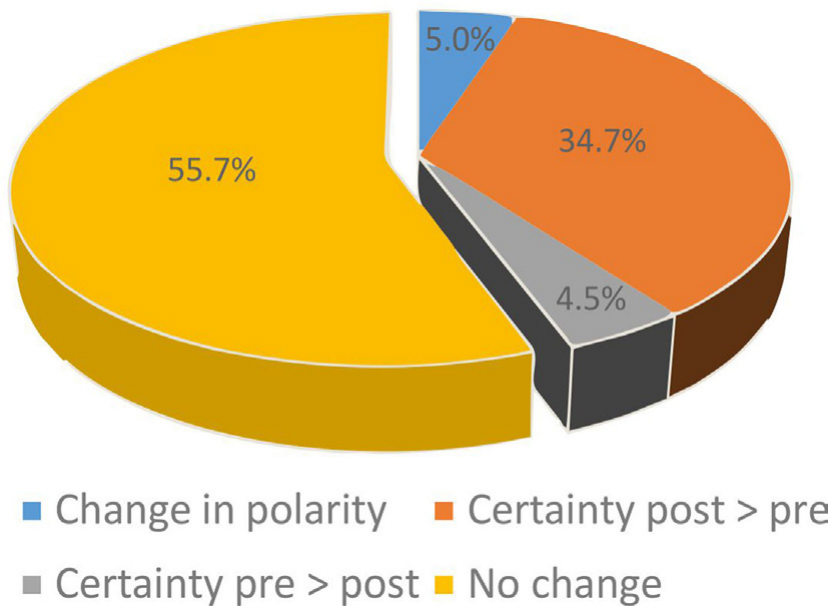
**Changes in certainty (blue, orange, gray) of bHIT after knowing the patient’s history by comparing pre- and post-bHIT**. The majority of these bHIT increased certainty from pre- to post-bHIT.

**Figure 2:**
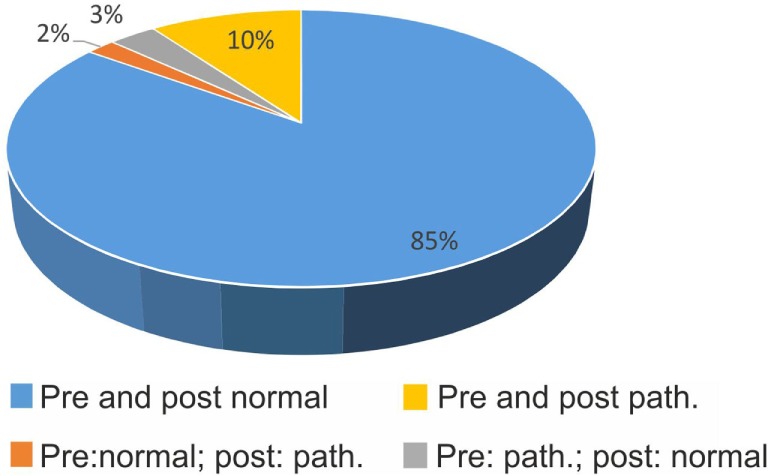
**Confirmation (blue, yellow) and changes (orange, gray) in polarity (pathological vs. normal) of bHIT before and after getting to know the patient’s history (pre- vs. post-bHIT)**.

In 18 patients, post-bHIT were not obtained by mistake. This reduced the number of pairs with pre- vs. post-bHIT to 993 bHIT (out of 1,030). Changes from pre- to post-bHIT occurred either at the level of certainty (normal or pathological, e.g., from 40 to 90% of certainty in calling a post-bHIT pathological) or in polarity (i.e., from pathological to healthy or *vice versa*). The bHIT assessment of 44.3% of all 993 post-bHIT (497 patients) changed after taking the patients’ history of vestibular symptoms (PVH): in 39.2% at the level of certainty (34.7% with an increase and 4.5% with a decrease in certainty in post-bHIT) (Figure 1) and in 5.0% of bHIT (*n* = 50) investigators changed polarity (e.g., from pathological to healthy) (Figure 2). The increase in confidence by the clinical investigators was largely due to changes from pre- to post-bHIT.

#### Direction of Changes

In 85%, clinical investigators did not change a normal (negative) bHIT result after PVH (Figure 2); in 10%, a pathological pre-bHIT stayed abnormal in post-bHIT. In 3%, pathological pre-bHIT was converted into a normal post-bHIT and, *vice versa*, in 2%, a normal pre-bHIT was changed to a pathological post-bHIT.

#### Accuracy of Changes

##### Does Knowing the Patients’ History Improve Correct Evaluations of Post-bHIT?

**Figure 3:**
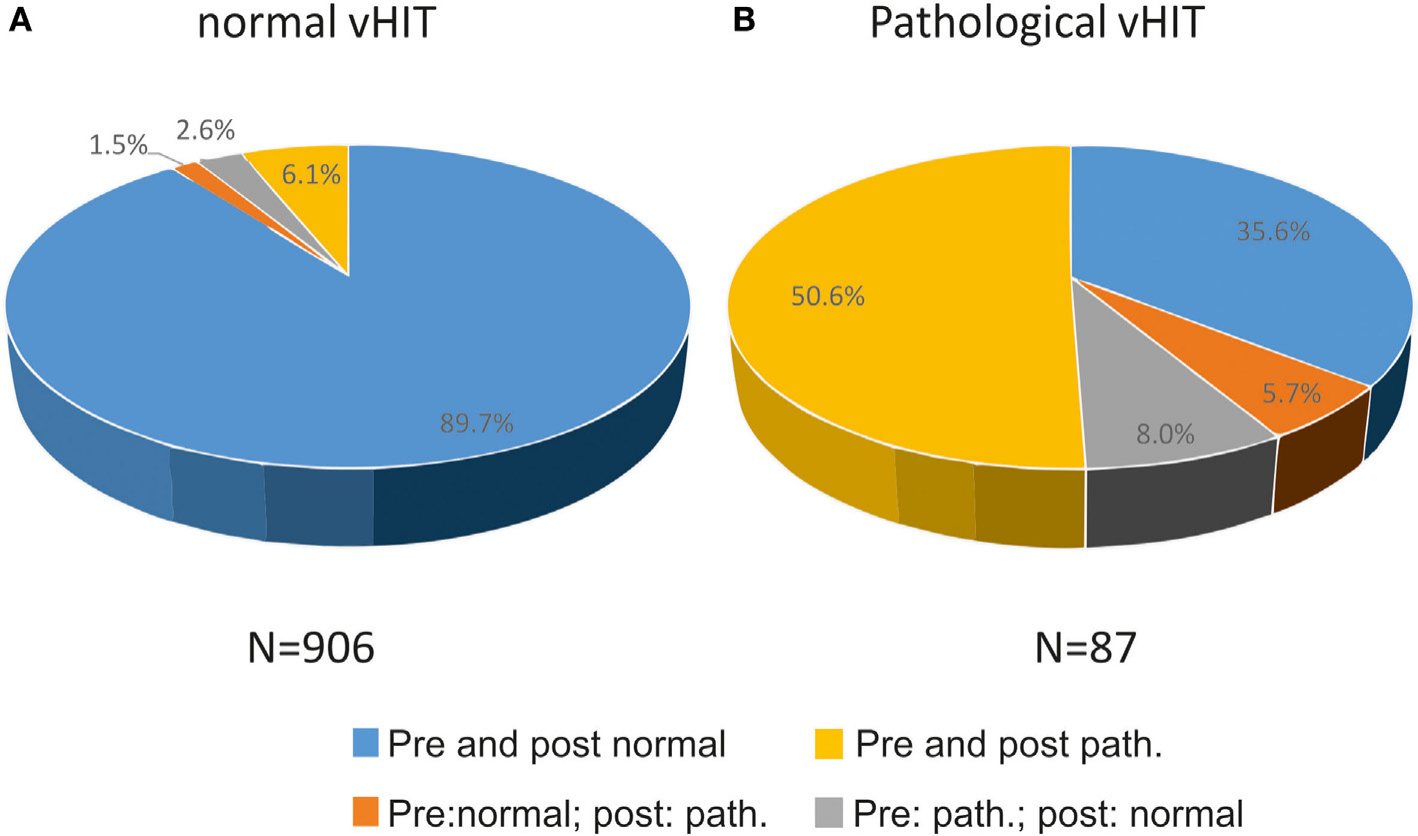
**Relation of changes (pathological to normal or vice versa) and confirmation of bHIT before and after getting to know the patient’s history to true normal and true pathological vHIT, i.e., 89.7% of all normal vHIT (***n*** = 906) were correctly rated normal before and after PVH (A)**. In contrast, 56.3% of patients were truly pathological on vHIT, either by confirming the pre-bHIT (yellow) or by changing from a normal to a pathological post-bHIT (orange) after knowing PVH **(B)**.

On quantitative testing, 90.8% of all vHIT were normal, 9.2% pathological. When compared with quantitative vHIT, the evaluation of a “normal” post-bHIT was correct in 92.3% which were rated normal on pre-bHIT, reflecting the very high NPV (Figure 3A). Thirty-eight (4.3%) normal post-bHITs were pathological on vHIT (false normal bHIT). Among all normal vHIT, 7.6% were rated as pathological post-bHIT.

By contrast, only 47 of 116 (40.5%) pathological post-bHIT showed truly pathological vHIT leading to the low PPV. Accordingly, 69 of 116 (59.5%) pathological post-bHIT were tested normal on vHIT (false pathological bHIT). Figure 3 shows the percentage of post-bHITs that were analyzed normal (Figure 3A) or pathological (Figure 3B) on vHIT. Among all pathological vHIT, 56.3% were rated as pathological bHIT, i.e., 43.6% of pathological vHIT were clinically classified as normal post-bHIT.

### Changes in Confidence (Certainty of Rating)

The level of certainty increased in 345 of all 993 bHIT (34.7%). Among those, the vast majority of increase in certainty after PVH was found when post-bHIT was classified and confirmed “*normal*” (288 of 356 = 80.9%), in fact 95.4% of normal bHIT with increased certainty on post-bHIT showed in fact a normal vHIT (Table 2). The increase in certainty in pathological post-bHIT, however, did not improve bHIT accuracy: only 17 (39.5%) of these 43 post-bHITs turned out to show pathological vHIT. In turn, in 60.5%, the increase in certainty of pathological post-bHIT misled to a false pathological post-bHIT as they showed normal vHIT (Table 2).

**Table 2:** **Changes in confidence (certainty of rating) from pre- to post-bHIT (without changes in polarity)**.

	Clinical rating after knowing patient’s history of vestibular symptoms	Quantitative vHIT			
		Normal		Pathological	
		*N*	Row (%)	*N*	Row (%)
No change	Normal	494	97.20	14	2.80
	Pathological	23	51.10	22	48.90
Change in polarity	Normal	24	77.40	7	22.60
	Pathological	14	73.70	5	26.30
Certainty post- > pre-bHIT	Normal	288	95.40	14	4.60
	Pathological	26	60.50	17	39.50
Certainty pre- > post-bHIT	Normal	31	91.20	3	8.80
	Pathological	6	54.50	5	45.50
Sum		906		87	

### Changes in Polarity

Polarity changes occurred in 50 post-bHITs (5%) in both directions, e.g., in 24 out of 31 post-bHIT (77.4%), the change from a pathological pre-bHIT to a normal post-bHIT was correct in vHIT. In turn, the vHIT was only pathological in 26.3% of pathological post-bHITs which were changed from a normal pre-bHIT (Table 2).

### Sensitivity of Post-bHIT in Bilateral Vestibular Failure

Twenty-nine (58 bHIT) patients showed bilateral vestibular failure (BVF) on vHIT. The mean VOR gain (at 60 ms after eye movement onset) in BVF patients was lower (mean gain: 0.41 ± 0.03; left = 0.37 ± 0.02, right = 0.44 ± 0.03) than in UVF (0.5 ± 0.04). However, sensitivity (56.3%) and specificity (92.4%) of post-bHIT did not differ from the values of all patients with vestibular failure.

### Clinical Expertise and Direction of Change in Post-bHIT

**Figure 4:**
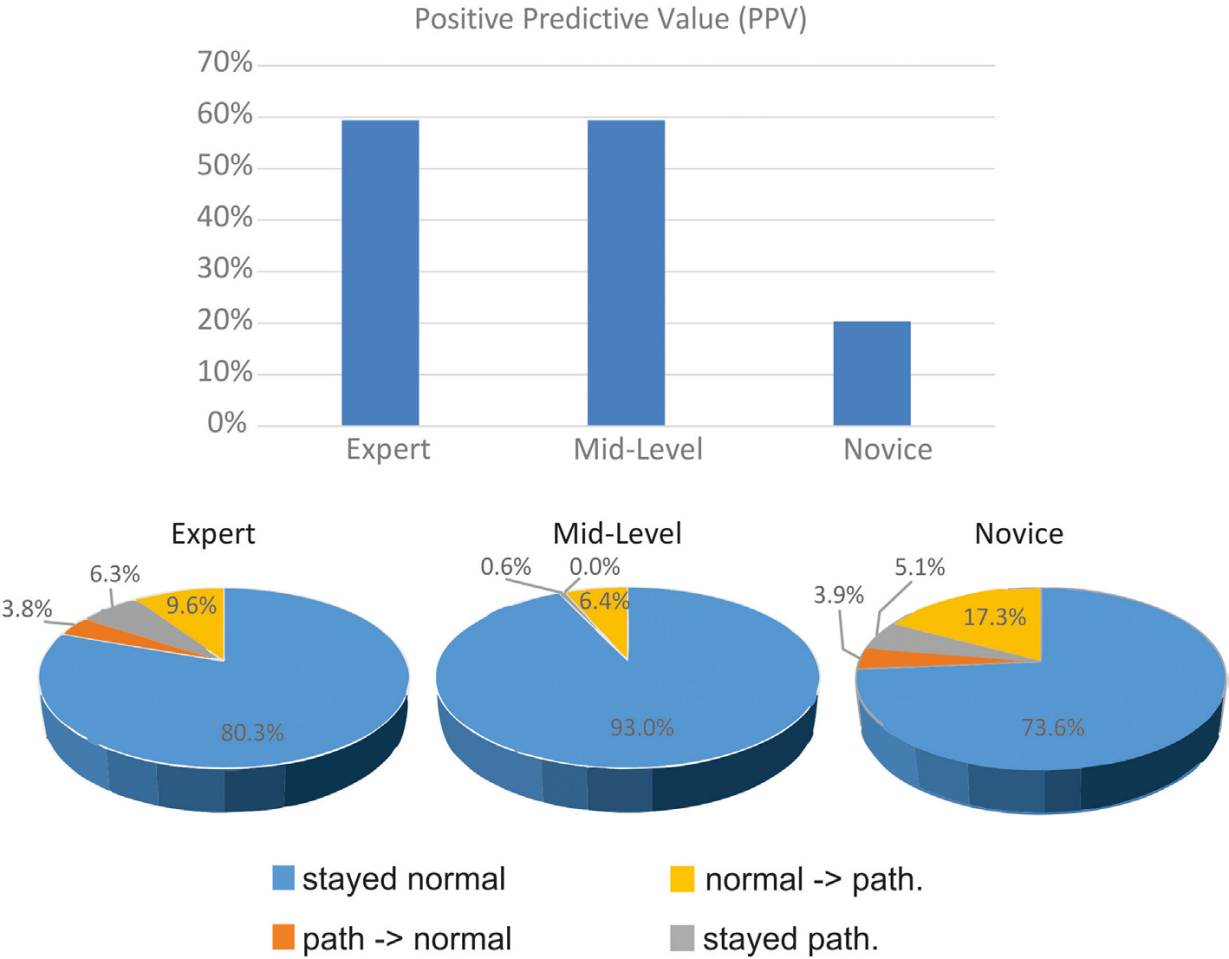
**Clinical evaluation of pre- and post-bHIT differs between clinicians with various expertise levels**. Positive predictive value of pre- and post-bHIT of the expert is much better than in novice clinicians.

Figure 4 displays the proportion of changes at different expert levels. It shows the large proportion of pathological pre- and post-bHIT in unexperienced novices. Only 9 out of 44 post-bHIT (20.5%) in novices were truly pathological on vHIT. Correct confirmation of pathological pre-BHIT was much higher in the experienced groups (mid-level: 59.4%; 69.6% in experts).

After PVH, the expert and novices switched from a pathological pre-bHIT to a normal post-bHIT in a similar proportion (expert: 6.3%, novices: 5.1%), in contrast to mid-level investigators who hardly changed at all (0.6%). In both groups, these changes were largely correct (novices: 92.3%, expert: 66.7%).

Only a few changes were made from a normal to a pathological post-bHIT (Figure 4; expert: 3.8% of all HIT, mid-level: 0%, novices: 3.9%): they were largely false in all groups (expert and mid-level: true pathological in 33.3%; novices: 20%).

Accordingly, the PPV differed with the level of clinical expertise (Figure 4): PPV of expert and mid-level investigators was much larger (59%) than in novices (11 of 54 bHIT, 20.4%). Sensitivity of novices was lower (47.8%) than the moderate sensitivity of the experienced groups (both 59.4%).

In contrast to sensitivity, specificity of unexperienced novices was much better but still lower (188 of 231 HITs, 81.4%) than in the experienced groups (mid-level: 97.2%, expert: 93.7%). NPV (94%) of novices did not differ from the experienced groups (expert: 93.7%; mid-level: 97.2%).

### Most Reliable Factor in Evaluating vHIT

The four raters, who analyzed the vHIT, rated the inspection of individual head and eye velocity trace profiles (“curves”) of vHIT in 60.1% (rater 1: 15.5%, rater 2: 70.2%, rater 3: 70.7%, rater 4: 83.9%) as the most helpful parameter in determining abnormal from normal HIT responses. The rater who did not use this method as frequently as the others preferred the so-called “Halmagyi gain” (rater 1: 51.1%, rater 2: 0.2%, rater 3: 0%, rater 4: 2.5%). The “gain at 60 ms” method was chosen on average in 3.8% and raters relied on corrective saccades in only on average 0.7%.

Despite differences between the raters in relying on the rating methods used, there was a high concordance between the raters’ vHIT assessment (pathological vs. normal) and one of the single gain determination methods: (A) gain at 60 ms (threshold gain <0.6:95.7%; <0.7:97.4%; <0.8:97.5%) and (B) “Halmagyi gain” (<0.6:92.3%; <0.7:93.1%; <0.8:91.2%). A cutoff gain of <0.7 is often taken pathological (7, 20).

Inter-rater reliability was high, tested by the Krippendorff’s alpha (0.92). Even without the exclusion of the 79 patients (ratings differed between raters, see Materials and Methods), Krippendorff’s alpha was still high (0.82). Cronbachs’ alpha was even higher (0.95) indicating the high overlap in ratings of the four independent raters.

## Discussion

Traditionally, clinicians in general consider taking a thorough history of patient’s symptoms crucial to establish the correct diagnosis. Specific symptoms (e.g., head movement-related dizziness) direct the clinician’s attention to the clinical examination of a particular (e.g., vestibular) organ. However, does history of organ-related symptoms improve the sensitivity and specificity of organ-related bedside examination in depicting its (mal)function? The knowledge of patients’ history may confirm or fool clinicians and increase or decrease the accuracy of bedside tests. We hypothesized that history of patient’s symptoms will improve the sensitivity of post-bHIT.

As a main result, almost half of all post-bHIT (46%) were changed by clinicians after obtaining PVH, either by changing the level of confidence or the polarity of post-bHIT. The changes in confidence led to the correct diagnosis (in vHIT) in the majority (95.4%) of normal post-bHIT. However, the increased level of confidence did not help in pathological post-bHIT evaluations. A polarity change (from pathological to normal and *vice versa*) was only found in 6% of all patients. The changes in polarity did overall not improve the correct evaluations of post-bHIT. However, they depended on the level of expertise since polarity changes of clinical experts revealed a greater proportion of correct post-bHIT.

The moderate sensitivity (60%) but consistently high specificity (90%) is in accord with previous studies testing bHIT with investigators being blinded for the patient’s history of vestibular symptoms (5, 7). The sensitivity of bHIT in bilateral vestibular failure (BVF) has been reported to be higher (84%) than in unilateral vestibular failure (23) which cannot be supported by our data.

Unlike hypothesized, PVH in our study did, on average, not change sensitivity and specificity of pre- and post-bHIT in UVF. However, this differed as a function of clinical neuro-otological experience in our clinical investigators. It is known that experts show a higher specificity (78%) but lower sensitivity (63%) of bHIT than non-experts (64 and 72%) (5). In our study, novice clinicians confirmed a pathological pre-bHIT much more often than the expert did. This led to the high incidence of false pathological findings in the unexperienced investigators. In turn, the expert changed a pathological pre-bHIT more often than the other groups and showed a higher rate of true pathological post-bHIT evaluations (73.1% as opposed to 18.5% in novice). In summary, experts use the knowledge of the patients’ history to increase the sensitivity of pathological bHIT and—at least partially—to correctly revise a pathological pre-bHIT to a truly normal post-bHIT (Table 3). They tend to change a previous pathological pre-bHIT only with clear hints of vestibular malfunction in the patient’s history and an increase in confidence. This is line with previous bHIT of experts being blinded for the patients’ history (5, 23). However, even the expert showed a significant number of false normal and pathological assignments in pathological vHIT.

**Table 3:** **Accuracy of pre- and post-bHIT in relation to video head-impulse test (vHIT)**.

When can I trust my bHIT?		
History of patient’s symptoms and bHIT		vHIT
bHIT before	bHIT after	Accuracy
Normal	Normal	+
Normal	Pathological	–
Pathological	Normal	(+) With expertise
Pathological	Pathological	–

*Recommendations for the use of vHIT are given for all conditions except for normal pre- and post-bHIT and a change from pathological to normal bHIT in experts*.

*+, accuracy is high; –, not reliable; (+), reliable in experts only*.

### Analytical Aspects

There are several approaches to analyze vestibular hypofunction. Procedural methods range from different time points (e.g., 60 ms after peak onset head acceleration) to intervals at peak head accelerations (20, 21), area under the desaccaded eye velocity curve during head movement (7), to corrective overt and covert saccades (24) the latter of which are hard to detect on clinical examination. Our four independent and blinded experienced HIT raters classified the inspection of traces of eye and head velocity as the most helpful sign in discriminating normal from pathological vHIT. Clinicians and neuro-otologists are advised to look at the original eye and head velocity curves rather than at refixation saccades alone or the gain at a given latency after head movement onset or interval which is highly variable depending on the performance, i.e., acceleration (11, 24).

The moderate sensitivity of bHIT has been shown to depend on the severity of unilateral vestibular failure and the magnitude of VOR asymmetries (5, 7): it increases with decreasing gain thresholds and larger VOR asymmetries. This was not our primary objective in this study, but for better comparability we compared our results with a cut-off gain often chosen in related studies on vestibular hypofunction (gain 0.7) (7, 20). As the confidence of our vHIT ratings highly correlated with this cut-off gain, we assume that our results on the effect of the PVH on bHIT are generalizable.

There are two limitations of the study. First, this study investigated patients with chronic vertigo symptoms. At present, it is unknown whether the results also hold for acute vestibular syndromes which inherently bear more risks of false evaluations both of bHIT and vHIT (19). Second, the results are confined to bHIT of the horizontal canals. Since there is less adaptation in vertical canals sensitivity of bHIT of vertical canals might increase, in particular in bilateral vestibular failure.

In conclusion, medical history of symptoms may lead to false diagnostic assumptions and may influence the clinical examination (bHIT) in false and correct directions. This first study on the effect of the investigators’ knowledge of the patient’s history of vestibular hypofunction on his clinical evaluation of the bHIT shows differential effects depending (i) on the state of disorder (normal vs. pathological) and (ii) the experience of the clinical investigator. Generally, irrespective of expertise level, the accuracy of normal post-bHIT was very high (NPV of 92.3%), which is higher than in previous related studies with the investigators blinded for the PVH (5, 23) but in line with a more recent study (7). Under pathological conditions, the accuracy of post-bHIT is only clinically reasonable in expert clinicians (sensitivity of 59%), which is not better than in previous studies with experts being blinded for PVH (5). In all other conditions (i.e., a change from normal to pathological and *vice versa*, pathological evaluations of post-bHIT in novices, Table 3), our data suggest the use of a quantitative vHIT, even in or despite of a positive PVH.

## Author Contributions

CH contributed to conceptualization and study design, project administration, data acquisition, supervision, drafting, editing, and approving the final writing of the manuscript. PT contributed to data acquisition and reviewing the manuscript. JK and AF contributed to methodology, data acquisition, and reviewing of the manuscript. AS contributed to study design, methodology, statistical analysis, reviewing, and editing of the manuscript.

## Conflict of Interest Statement

The authors declare that the research was conducted in the absence of any commercial or financial relationships that could be construed as a potential conflict of interest.

## References

[1] Halmagyi GM Curthoys IS. A clinical sign of canal paresis. Arch Neurol (1988) 45:737–9. 10.1001/archneur.1988.00520310043015 3390028

[2] MacDougall HG McGarvie LA Halmagyi GM Curthoys IS Weber KP. The video head impulse test (vHIT) detects vertical semicircular canal dysfunction. PLoS One (2013) 8:e61488. 10.1371/journal.pone.0061488 23630593 PMC3632590

[3] Agrawal Y Schubert MC Migliaccio AA Zee DS Schneider E Lehnen N Evaluation of quantitative head impulse testing using search coils versus video-oculography in older individuals. Otol Neurotol (2014) 35:283–8. 10.1097/MAO.0b013e3182995227 24080977 PMC4532669

[4] Heuberger M Sağlam M Todd NS Jahn K Schneider E Lehnen N. Covert anti-compensatory quick eye movements during head impulses. PLoS One (2014) 9:e93086. 10.1371/journal.pone.0093086 24732783 PMC3986070

[5] Jorns-Haderli M Straumann D Palla A. Accuracy of the bedside head impulse test in detecting vestibular hypofunction. J Neurol Neurosurg Psychiatry (2007) 78:1113–8. 10.1136/jnnp.2006.109512 17220287 PMC2117540

[6] Bartolomeo M Biboulet R Pierre G Mondain M Uziel A Venail F. Value of the video head impulse test in assessing vestibular deficits following vestibular neuritis. Eur Arch Otorhinolaryngol (2014) 271:681–8. 10.1007/s00405-013-2451-y 23539412

[7] Yip CW Glaser M Frenzel C Bayer O Strupp M. Comparison of the bedside head-impulse test with the video head-impulse test in a clinical practice setting: a prospective study of 500 outpatients. Front Neurol (2016) 7:58. 10.3389/fneur.2016.00058 27148159 PMC4837142

[8] Matino-Soler E Rey-Martinez J Trinidad-Ruiz G Batuecas-Caletrio A Perez Fernandez N. A new method to improve the imbalance in chronic unilateral vestibular loss: the organization of refixation saccades. Acta Otolaryngol (2016) 136:894–900. 10.3109/00016489.2016.1172730 27109262

[9] Perez-Fernandez N Gallegos-Constantino V Barona-Lleo L Manrique-Huarte R. Clinical and video-assisted examination of the vestibulo-ocular reflex: a comparative study. Acta Otorrinolaringol Esp (2012) 63:429–35. 10.1016/j.otorri.2012.04.010 22789453

[10] Strupp M Dieterich M Zwergal A Brandt T. [Diagnosis and treatment options in vertigo syndromes]. Nervenarzt (2015) 86:1277–90. 10.1007/s00115-015-4389-3 26440631

[11] Machner B Sprenger A Füllgraf H Trillenberg P Helmchen C. [Video-based head impulse test. Importance for routine diagnostics of patients with vertigo]. Nervenarzt (2013) 84:975–83. 10.1007/s00115-013-3824-6 23839059

[12] Straumann D Bedside examination. Handb Clin Neurol (2016) 137:91–101. 10.1016/B978-0-444-63437-5.00007-8 27638065

[13] Göttlich M Jandl NM Sprenger A Wojak JF Münte TF Krämer UM Hippocampal gray matter volume in bilateral vestibular failure. Hum Brain Mapp (2016) 37:1998–2006. 10.1002/hbm.23152 26918638 PMC6867258

[14] Bartl K Lehnen N Kohlbecher S Schneider E. Head impulse testing using video-oculography. Ann N Y Acad Sci (2009) 1164:331–3. 10.1111/j.1749-6632.2009.03850.x 19645921

[15] Göttlich M Jandl NM Wojak JF Sprenger A von der Gablentz J Münte TF Altered resting-state functional connectivity in patients with chronic bilateral vestibular failure. Neuroimage Clin (2014) 4:488–99. 10.1016/j.nicl.2014.03.003 24818075 PMC3984447

[16] Petersen JA Straumann D Weber KP. Clinical diagnosis of bilateral vestibular loss: three simple bedside tests. Ther Adv Neurol Disord (2013) 6:41–5. 10.1177/1756285612465920 23277792 PMC3526948

[17] Schneider E Villgrattner T Vockeroth J Bartl K Kohlbecher S Bardins S EyeSeeCam: an eye movement-driven head camera for the examination of natural visual exploration. Ann N Y Acad Sci (2009) 1164:461–7. 10.1111/j.1749-6632.2009.03858.x 19645949

[18] Halmagyi GM Black RA Thurtell MJ Curthoys IS. The human horizontal vestibulo-ocular reflex in response to active and passive head impulses after unilateral vestibular deafferentation. Ann N Y Acad Sci (2003) 1004:325–36. 10.1196/annals.1303.030 14662472

[19] Mantokoudis G Saber Tehrani AS Kattah JC Eibenberger K Guede CI Zee DS Quantifying the vestibulo-ocular reflex with video-oculography: nature and frequency of artifacts. Audiol Neurootol (2015) 20:39–50. 10.1159/000362780 25501133

[20] MacDougall HG Weber KP McGarvie LA Halmagyi GM Curthoys IS. The video head impulse test: diagnostic accuracy in peripheral vestibulopathy. Neurology (2009) 73:1134–41. 10.1212/WNL.0b013e3181bacf85 19805730 PMC2890997

[21] McGarvie LA MacDougall HG Halmagyi GM Burgess AM Weber KP Curthoys IS. The video head impulse test (vHIT) of semicircular canal function – age-dependent normative values of VOR gain in healthy subjects. Front Neurol (2015) 6:154. 10.3389/fneur.2015.00154 26217301 PMC4495346

[22] Hayes AF Krippendorff K Answering the call for a standard reliability measure for coding data. Commun Methods Meas (2007) 1:77–89. 10.1080/19312450709336664

[23] Schubert MC Tusa RJ Grine LE Herdman SJ. Optimizing the sensitivity of the head thrust test for identifying vestibular hypofunction. Phys Ther (2004) 84:151–8. 10.1093/ptj/84.2.151 14744205

[24] Weber KP Aw ST Todd MJ McGarvie LA Curthoys IS Halmagyi GM. Head impulse test in unilateral vestibular loss: vestibulo-ocular reflex and catch-up saccades. Neurology (2008) 70:454–63. 10.1212/01.wnl.0000299117.48935.2e 18250290

